# First record of *Graphosoma inexpectatum* (Hemiptera, Pentatomidae, Podopinae) from Turkey with description of the female

**DOI:** 10.3897/zookeys.319.4298

**Published:** 2013-07-30

**Authors:** Meral Fent, Ahmet Dursun, Serdar Tezcan

**Affiliations:** 1 Trakya University, Faculty of Science, Department of Biology, 22030 Edirne, Turkey; 2 Amasya University, Faculty of Arts and Science, Department of Biology, 05100 Amasya, Turkey; 3 Ege University, Faculty of Agriculture, Department of Plant Protection, 35100 Bornova İzmir, Turkey

**Keywords:** Hemiptera, Pentatomidae, Podopinae, *Graphosoma inexpectatum*, female description, first record, Turkey

## Abstract

*Graphosoma inexpectatum* Carapezza & Jindra, 2008 is described from Syria, the southern neighbor of Turkey, and is known only from the type locality. The first observation of the species in Turkey dates back to 1995 with two females obtained from the provinces of Gaziantep (Şehitkamil–Aktoprak) and Adana (Pozantı–Bürücek Plateau). These two localities are situated inside the part of the Mediterranean region along the Syrian border. Females of the species, whose original description was based on males, are described here for the first time. A map showing the collecting localities and photographs of the female specimens are given.

## Introduction

*Graphosoma* Laporte, 1833 is a Palaearctic genus belonging to the subfamily Podopinae Amyot & Serville, 1843 of the family Pentatomidae Leach, 1815. It is subdivided into two subgenera and includes nine species/subspecies (*Graphosoma* s.str. with eight species and *Graphosomella* Carapezza & Jindra, 2008 with one species). Among these nine species, *Graphosoma inexpectatum* Carapezza & Jindra, 2008, the only representative of *Graphosomella*, and the following six species of *Graphosoma* s.str. are distributed in Turkey: *Graphosoma lineatum* (Linnaeus, 1758), *Graphosoma semipunctatum* (Fabricius, 1775), *Graphosoma melanoxanthum* Horváth, 1903, *Graphosoma stali* Horváth, 1881, *Graphosoma consimile* Horváth, 1903 and *Graphosoma alkani* Lodos, 1959 (Rider 2006, Péricart 2010).

Among the Turkish representatives of the genus, the type localities of *Graphosoma stali* and *Graphosoma alkani* are in Turkey. *Graphosoma stali* was previously identified as *Graphosoma lineatum* var. *stali* by Horváth in 1881 from Asia Minor (Rider 2006). In the following years, the species was recorded in various parts of Anatolia; from Hatay–Akbez (Puton 1896, Horváth 1903) along the Syrian border and in Gaziantep (Önder et al. 1995) in the south, from Mardin (Horváth 1903, Önder et al. 1995) and Diyarbakır (Wagner 1959, Önder et al. 1995, Özgen et al. 2005) in the southeast and from Kars–Kağızman, Muş–Dom (Kiritshenko 1924) and Elazığ (Hazar Lake) in eastern Anatolia (Seidenstücker 1975). *Graphosoma stali* is a Mediterranean species whose distribution includes eastern and southern neighbors of Turkey—Iran, Iraq and Syria—and also Israel. *Graphosoma alkani* is an endemic species described by Lodos (1959) from Diyarbakır and Mardin in southeastern Anatolia. Both Carapezza and Jindra (2008) and Péricart (2010) expressed the opinion that *Graphosoma alkani* might be a junior synonym of *Graphosoma stali*. *Graphosoma lineatum* and *Graphosoma semipunctatum* are both widely distributed in the Mediterranean region and have been recorded in a number of localities in European (Turkish Thrace) and Asian (Anatolia) parts of Turkey (Önder et al. 2006). In contrast to the wide Palaearctic distribution of *Graphosoma lineatum*, the range of *Graphosoma semipunctatum* is limited to the Mediterranean region, Caucasus and Transcaucasia (Rider 2006, Péricart 2010). *Graphosoma melanoxanthum* was recorded inIğdır–Tuzluca (=Kulp), Kazkoparan (=Kasikoparan) (Horváth 1903) and Kars–Sarıkamış (Kiritshenko 1918) along the Armenian border in eastern Anatolia, in Elazığ–Maden in more inner parts (Kıyak 1990), in Ankara–Kızılcahamam in Central Anatolia (Seidenstücker 1975) and in Yalova in northwestern Anatolia (Lodos et al. 1978). This species was also recorded, in addition to Anatolia, within an area including Georgia, Armenia, Azerbaijan and Iran, the northeast and east neighbors of Turkey (Rider 2006, Péricart 2010). *Graphosoma consimile* was reported in Anatolia from Kayseri–Yılanlı Dağ (Yılanlı Mountain) by Seidenstücker (1975) and from the vicinity of Elazığ–Hazar Lake by Kıyak (1990). The range of this Asian species includes Afghanistan, Azerbaijan, Georgia, Iran, Kazakhstan, Tajikistan, Turkmenistan and Uzbekistan (Rider 2006, Péricart 2010).

*Graphosoma* (s.str.) *interruptum* A. White, 1839 distributed only on the Canary Islands and *Graphosoma* (s.str.) *rubrolineatum* (Westwood, 1837) distributed in the Far East (China, Japan, Korea, Mongolia and Russian Far East) do not occur in Turkey (Rider 2006, Péricart 2010).

*Graphosoma (Graphosomella) inexpectatum* Carapezza & Jindra, 2008, the only species of the subgenus *Graphosomella* Carapezza & Jindra, 2008, is described from Syria (type locality: SW Syria, Bludan) based on two male specimens and has not been recorded in any other place so far. Two females were obtained during the present study from Adana and Gaziantep provinces, which are both very close to Syria.

## Material and methods

The study material was collected in June–July of 1995 in two southern provinces of Turkey, Gaziantep (Şehitkamil–Aktoprak) and Adana (Pozantı–Bürücek Plateau), using a sweeping net. Aktoprak is a district of the city Şehitkamil. This area, located on the border between the southeastern Anatolian and Mediterranean regions of Turkey, has a transition climate which includes both Mediterranean and continental climate characteristics. Winters in the area are cold and wet, and summers are hot and dry. As a result of the climate, the flora of this territory is transitional between the vegetation types of the Mediterranean and the steppe elements of the southeastern Anatolian regions. Although forests are rare, the dominant trees oak (*Quercus* sp.) and red pine (*Pinus brutia* Ten.) were chosen in forested areas (www.markasehir.com/siteic.php~id=&altno=23&back=false.html).

The second locality, Bürücek Plateau, is 100 km away from Adana city center and is an upland with an altitude of 1300 m surrounded by pines (*Pinus* spp.), junipers (*Juniperus* spp.) and fruit trees at the foot of the Akdağ Mountains, Middle Taurus. Pozantı is 7 km away from the Bürücek Plateau and is under the effect of a Mediterranean climate with cold and wet winters and hot and dry summers. Snowfall is typical for winter, and rainfall, during the spring. Despite the hot weather conditions in spring and autumn, Bürücek Plateau is cool even in summer. The area is characterized by the Mediterranean phytogeographical region’s vegetation formations under the influence of the climate. The dominant tree of the upland is generally the red pine (*Pinus brutia* Ten.) but the amount of mixed forest areas including both black pines (*Pinus nigra* Arnold) and red pines increases with higher altitudes; at higher altitudes black pine, cedar (*Cedrus libani* A. Rich.) and fir (*Abies cilicica* Carr.) formations exist in either mixed or pure areas. In addition, trees such as Syrian juniper (*Juniperus drupacea* Lab.), ash (*Fraxinus excelsior* L.), oak (*Quercus* sp.), willow (*Salix* sp.), hornbeam (*Carpinus betulus* L.), European cornel (*Cornus mas* L.), European bladdernut (*Staphylea pinnata* L.), hawthorn (*Crataegus* sp.), blackberry (*Rubus fruticosus* L.), service tree (*Sorbus domestica* L.) and spruce (*Picea* sp.) and annual plants such as St. John’s worth (*Hypericum perforatum* L.), oregan (*Origanum vulgare* L.), thyme (*Thymus vulgaris* L.), speedwell (*Veronica officinalis* L.), wild garlic (*Alium ursinum* L.), chard (*Beta vulgaris* var. *cicla* L.), salep (*Orchis mascula* L.), horsemint (*Mentha longifolia* L.) and colchicum(*Colchicum autumnale* (L.)) are commonly seen (www.adanaliyiz.org/index.php?topic=48.0;wap).

## Results

### Graphosoma (Graphosomella) inexpectatum

Carapezza & Jindra, 2008

http://species-id.net/wiki/Graphosoma_inexpectatum

#### Material examined.

Gaziantep province: Şehitkamil (Aktoprak), 11.VI.1995, 37°11'00"N, 37°17'00"E, ca 1035 m, 1 ♀, leg. F. Önder; Adana province: Pozantı (Bürücek Plateau), 2.VII.1995, 37°25'40"N, 34°52'18"E, ca 1300 m, 1 ♀, leg. F. Önder (coll. Trakya University, Edirne, Turkey and Ege University, LEMT, İzmir, Turkey).

#### Host plants.

The host plants for both specimens were recorded as weeds, so the plant on which specimens were collected is not known exactly.

#### Description of female

**Figure 1.**
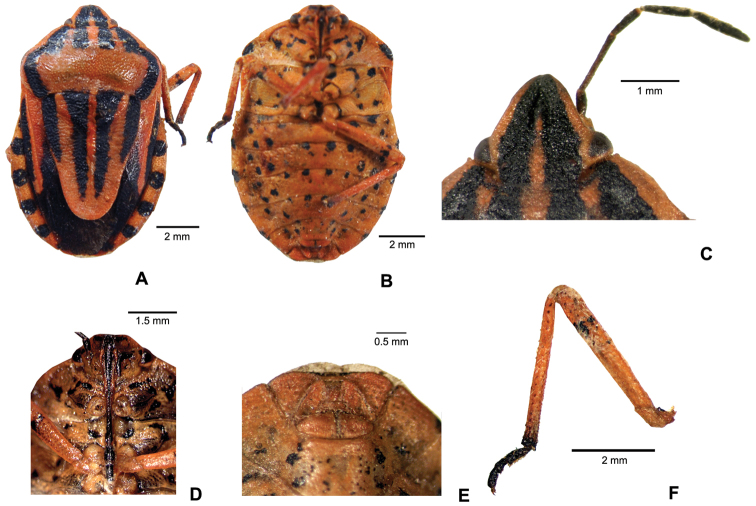
*Graphosoma inexpectatum*. **A** body, dorsal view **B** body, ventral view **C** head, dorsal view, and antenna **D** head, ventral view, and rostrum **E** female genitalia **F** leg, lateral view.

(Fig. 1). Body ovoid, flat, moderately deep punctate and glabrous. Coloration pattern of body with black lines and markings on orange as in most species of *Graphosoma*. Body 1.75–1.80× as long as pronotum width. Body is slightly greater than in males (10.3–11.3 mm versus 10.66–10.80 mm) (Fig. 1A).

Head almost subtriangular, lateral sides nearly flat. Head: 1.77–1.99 mm long and 0.76–0.82× longer than wide across eyes; width across eyes 2.32–2.41 mm, interocular width 1.52–1.65 mm; jugae enclosing and widely exceeding the tylus, slightly diverging apically. Dorsal surface of head deeply punctate. Integument orange, with two black bands tapering and fusing distally. Length of antennomeres I: 0.54–0.62 mm, II: 1.0–1.13 mm, III: 0.64–0.66 mm, IV: 0.7–0.9 mm, V: 0.95–1.06 mm. First and second antennomeres are the shortest and the longest respectively. First antennomere brownish, yellowish distally; IV and V antennomeres dark brown (Fig. 1C). Antennae/Body length: 0.37–0.38. Head ventrally orange, anterior angle of eye with small black subtriangle spot, jugae anteroventrally with transverse black spot (Fig. 1D).

Pronotum transverse, 5.87–6.29 mm wide across lateral angles and 1.92–2.22× wider than long in the middle. Pronotum anteriorly rather narrower than posteriorly, lateral margins evenly rounded. Dorsal surface of pronotum deeply punctate and orange, anterior part with four longitudinal black bands, the external ones at their posterolateral angles joining two curved black bands running parallel to posterior half of lateral pronotal margin (Fig. 1A).

Scutellum subtriangular, distally widely rounded, 5,4–6,3 mm long and 1.28–1.34× longer than basally wide; contrary to species of *Graphosoma* s.str., scutellum does not reach to the end of abdomen and covers only two thirds of abdomen length. Lateral margins of scutellum convex in proximal half. Proximal margin of scutellum medially with a raised semicircular punctureless area between the internal margins of two lateral black bands; lateral margins of semicircular area extend to internal margins of lateral bands. Scutellum orange with four long black bands; lateral bands shorter than median ones, distally almost pointed and extending to half length of scutellum; median bands long but not reaching the apex of scutellum (Fig. 1A).

Hemelytra orange except for a triangular blackish brown spot at the apex of r+m vein, the external margin of exocorium with longitudinal black spot, distal with triangular spot, membran blackish–brown (Fig. 1A).

Paratergites with black spots along distal and proximal margins, spots of adjoining tergites merging to form an almost circular shape (Fig. 1A).

Abdomen maximum width 6.88–6.99 mm and 1.09–1.17× wider than long, ventrally orange and with irregular black spot. Proximal and distal angles of parasternites with black spots (Fig. 1B).

Rostrum reaching hind coxae; first three segments yellowish, IV dark brown (Fig. 1D). Length of rostral segments I–IV: 1.56, 1.13, 0.82, 0.53 mm.

Legs orange, femora ventrally with two preapical black spots, apex of tibiae black, tarsi black (Fig. 1F).

Female genitalia reddish–brown, with shallow punctures. Eight gonocoxites convex, 9^th^ gonocoxites medially excavated, lateral margins with black lines and centrally with black mark (Fig. 1E).

#### Comparative notes.

The two female specimens examined in the present study show some differences in morphology and coloration when compared with the holotype of the species whose original description by Carapezza and Jindra (2008) was based on two male specimens. The antennomeres (I to V) of the holotype are 0.6 / 1.06 / 0.53 / 0.73 / 1.06 mm long, hence the shortest segment is III. Antennomeres II and V are of equal length, I is a bit longer than III, and IV is longer than I. Antennomeres of Turkish specimens (I to V) are (0.54–0.62), (1.0–1.13), (0.64–0.66), (0.7–0.9), (0.95–1.06) mm long. First and III antennomeres are the shortest with somewhat equal lengths and II is the longest. The ratio of antennal length to body length is 0.33 in the holotype and 0.37–0.38 in our specimens (Fig. 1C). The bands in the middle of the scutellum of the holotype are wide basally and gradually taper towards the middle of the scutellum and reach the end as thin bands. However, the narrowing after the middle in our specimens is not pronounced and bands are comparatively wide (Fig. 1A). Moreover, the black spots seen on proximal and distal margin of each paratergite are shaped as black bands in the holotype and the distal band of one segment and the proximal band of the next segment join to form a rectangular shape. These black spots on each segment are in semicircular shape in our specimens and form an almost circular shape when joined (Fig. 1A). Since the comparison was carried out between the males from Syria and the females from Turkey, it is impossible to conclude if the differences put into evidence depend on sexual dimorphism or on geographical variations.

## Discussion

Two female specimens of *Graphosoma (Graphosomella) inexpectatum* were obtained in 1995 from two localities in Adana and Gaziantep provinces in the Mediterranean region of Turkey. This is the first record of the species in Turkey and the second record in the world. The number of *Graphosoma* species in Turkey rises to seven with this record. The presence of seven species of *Graphosoma* in Anatolia, out of the nine overall known in the genus, reinforces the notion that Anatolia and its immediate vicinities, as indicated by Carapezza and Jindra (2008), might be the center of origin of the genus.

**Figure 2.**
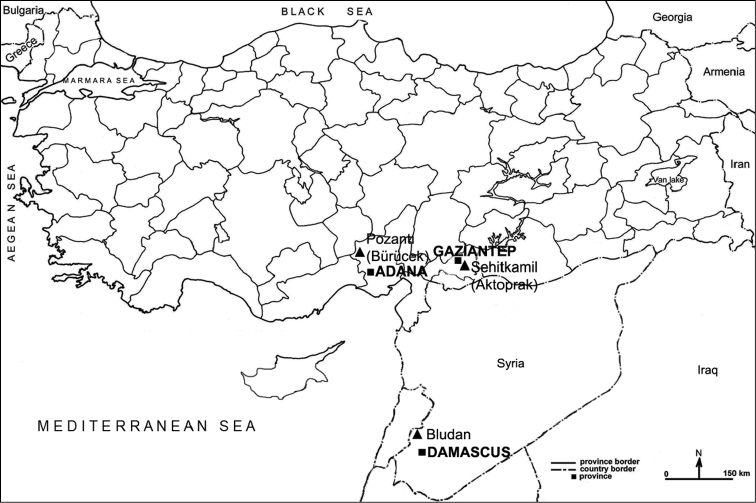
Distribution of *Graphosoma inexpectatum*.

This species was first described by Carapezza and Jindra (2008) from Bludan in SW Syria. Bludan is a locality situated in a mountain valley (1590–2100 m a.s.l.) with relatively rich steppe vegetation in the Anti–Lebanon Mountains, about 30 km NW of Damascus (Carapezza and Jindra 2008). The localities in Turkey are close to Syria and the presence of all currently known localities of the species in Mediterranean Region supports the possibi-lity that the species is a Syrian–Anatolian element of east Mediterranean origin (Fig. 2).

## Supplementary Material

XML Treatment for Graphosoma (Graphosomella) inexpectatum
